# The Effect of Acromioplasty Performed During Rotator Cuff Repairs on Clinical Outcomes in Patients With Type 3 Acromion: A Retrospective Study

**DOI:** 10.7759/cureus.48867

**Published:** 2023-11-15

**Authors:** Seçkin Özcan, Hakan Yurten

**Affiliations:** 1 Orthopedics and Traumatology, Yalova Education and Research Hospital, Yalova, TUR; 2 Orthopedics and Traumatology, Ministry of Health Elaziğ Fethi̇ Seki̇n City Hospital, Elazığ, TUR

**Keywords:** impingement, type 3 acromion, subacromial distance, rotator cuff, acromioplasty

## Abstract

Objective: Rotator cuff (RC) tears often necessitate surgery, with acromioplasty being frequently performed alongside RC repair. However, the impact of acromioplasty on clinical outcomes remains a subject of discussion. The current study aimed to investigate the effect of acromioplasty during RC repair on clinical outcomes in patients with type 3 acromion.

Materials and methods: Eighty-five patients, who underwent RC repair between January 2016 and December 2020, were categorized into two groups: Group 1, comprising 40 patients without acromioplasty, and Group 2, including 45 patients who received acromioplasty. Subacromial distance (SAD) and clinical scores were assessed pre- and post-operatively.

Results: Group 1, comprising 40 patients without acromioplasty, had an average age of 59.45±10.43 years. Among them, 27 (67.5%) were female. The mean symptom duration was 10.4±4.3 months, and the mean follow-up period was 16.2±1.9 months. Group 2, which underwent acromioplasty, included 45 patients with an average age of 56.49±8.97 years, with 30 (66.7%) of them being female. The mean symptom duration was 9.5±3.6 months, and the mean follow-up time was 15.78±2.17 months in this group. Group 2 showed a relatively greater improvement in SAD compared to Group 1. The evaluation of clinical outcomes did not reveal any significant differences between the groups.

Conclusion: Concomitant acromioplasty does not have a significant effect on clinical outcomes in patients undergoing arthroscopic RC repair.

## Introduction

Rotator cuff tears (RCTs) are frequently encountered by orthopaedic surgeons, driven by an increase in physically active elderly individuals and extended life expectancy [1-3]. While affecting approximately 30% of the population aged 60 and older, the incidence of RCTs rises with advancing age [4]. When planning the treatment for RCTs, factors such as tear location, size, associated lesions, and etiological considerations are taken into consideration. With the growing number of RC repair surgeries, numerous studies have been conducted to explore the etiological factors [1-4].

Until recently, extrinsic factors were emphasized as contributors to RCT. These factors such as the anatomical shape of the acromion or the subacromial spur were thought to restrict the movements of the rotator muscles by narrowing the subacromial space, thus resulting in subacromial pain syndrome and RCT. For this reason, it has become increasingly common to perform acromioplasty with RC repair to address these factors by enlarging the subacromial space [1,2,5]. However, contemporary research suggests that extrinsic factors may not have a significant effect as previously claimed. Current studies have shifted their focus more toward intrinsic etiological factors [6]. It is thought that the accumulation of any damage within the tendon reduces its resistance, particularly against eccentric forces, ultimately leading to RCT. These studies advocated that acromioplasty may not yield meaningful improvements in clinical outcomes [6,7].

There is a dynamic relationship between the RCT and the subacromial distance (SAD), which is determined by measuring the distance between the lower border of the acromion and the humeral head. A narrow SAD is believed to contribute to RCT. When the RC is damaged, its compressive effect on the humeral head diminishes, causing superior migration of the humeral head and reduction in SAD [8,9]. This study aimed to investigate the impact of acromioplasty in patients with type 3 acromion who have undergone arthroscopic RC repair. Our hypothesis posits that acromioplasty may not significantly affect the clinical outcomes of arthroscopic RC repair in patients with full-thickness RCT and type 3 acromion.

## Materials and methods

Study population

**Figure 1:**
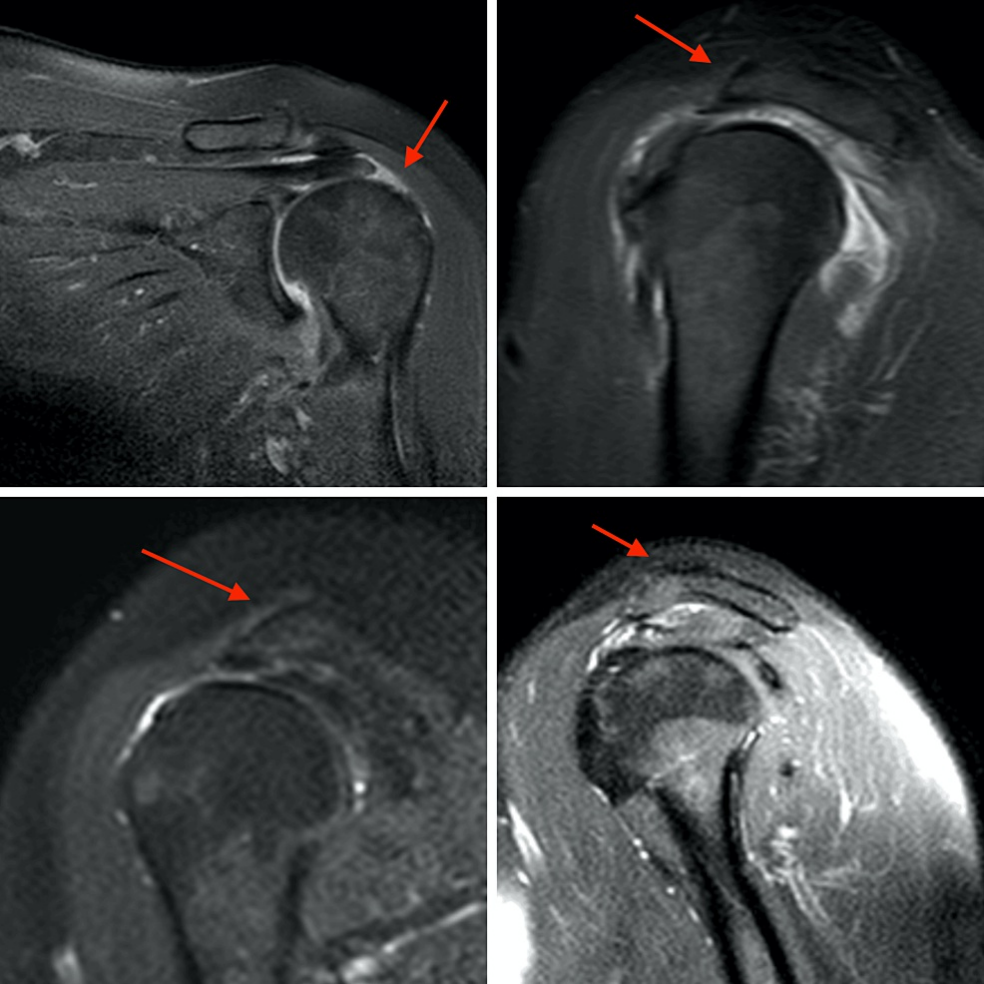
Magnetic resonance images of RCT and type 3 acromion. RCT: Rotator cuff tear

This retrospective case-control study was approved by our institution’s ethical review board and conducted in accordance with the Declaration of Helsinki. Patients with type 3 acromion, whose diagnosis of full-thickness RCT was confirmed clinically and radiologically and who underwent arthroscopic repair between January 2016 and December 2020 were included in the study (Figure 1).

A total of 96 patients received treatment within this timeframe. Exclusion criteria encompassed incomplete follow-up or inadequate medical records (n:5), partial-thickness or irreparable full-thickness RCTs (Patte Classification Stage 3 or Goutallier Classification Grade ≥3) (n:4), and history of upper extremity surgery or fracture (n:2). Patients who had a full-thickness RCT but required secondary repair, such as those with a tear in the subscapularis muscle or a slap lesion were also excluded. Eighty-five patients (28 males, 57 females) included in our study were categorized into two groups: Group 1, comprising 40 patients without acromioplasty, and Group 2, which included 45 patients who received acromioplasty.

Surgical technique

**Figure 2:**
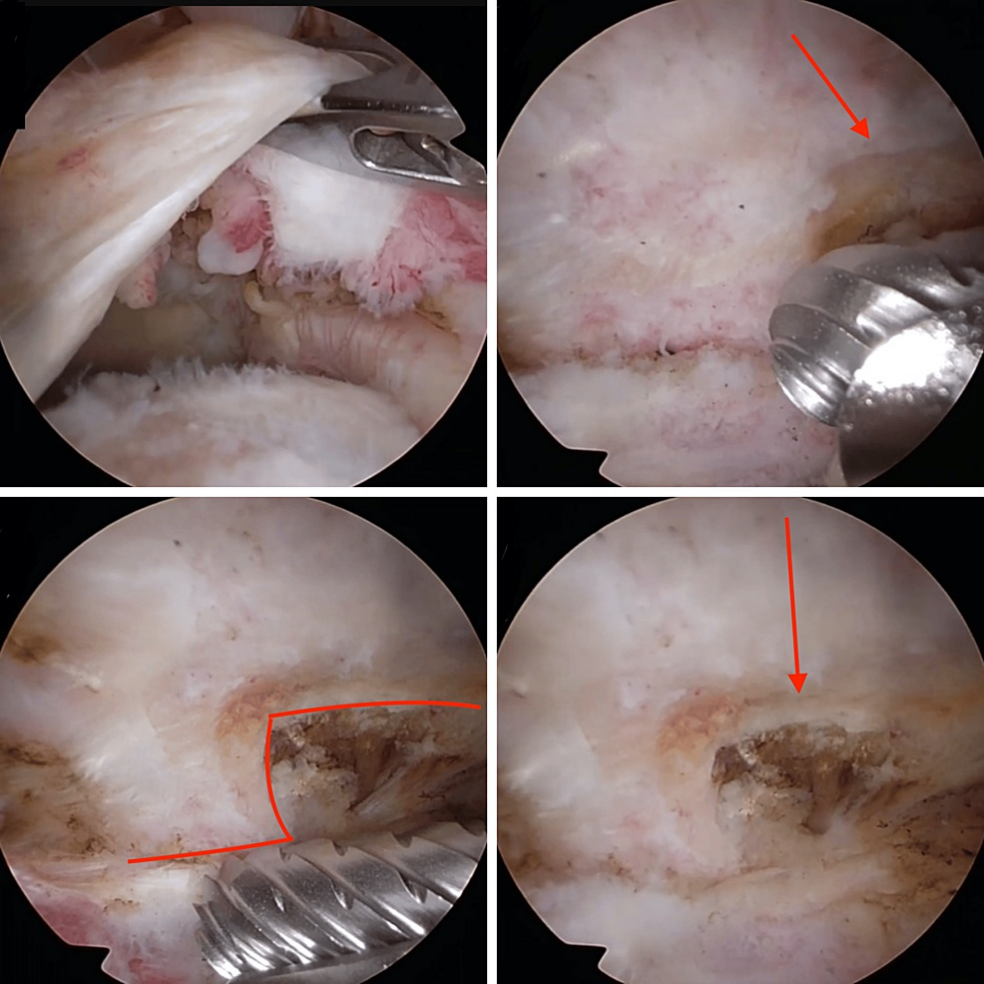
Arthroscopic images from the acromioplasty procedure

All patients received comprehensive pre-surgical information and provided informed consent. Two senior surgeons in our department performed all procedures simultaneously, with patients positioned in the lateral decubitus position. Standard glenohumeral and subacromial arthroscopy was performed. In Group 2, following arthroscopic RC repair, the arthroscope was inserted from the standard posterior portal. Acromioplasty was performed with a burr placed from the lateral portal, creating a smooth surface at the posterior part of the acromion and deepening by 0.5 cm at the anterior end (Figure 2).

All patients initiated passive shoulder motion and pendulum exercises one week after the surgery. This practice was continued for the postoperative initial six-week period. After this first six-week period, a standard physical therapy program was implemented, including more aggressive passive and active shoulder movements and muscle strengthening. Patients were regularly called to the outpatient clinic for follow-up visits and their physical examinations were repeated. All patients were compliant with follow-up and physical therapy programs.

Data collection

Patients were evaluated for functional and clinical outcomes at preoperative and 12-month postoperative. All patients were evaluated by the same surgeon during the preoperative and postoperative periods. At the time of initial admission, patient data including age, gender, and symptom duration (in months) were recorded. SAD measurements, both in sagittal (sSAD) and coronal (cSAD) planes, were conducted using MRI by an observer who had experience in evaluating musculoskeletal system images. Shoulder range of motion (ROM) measurements, visual analogue scale (VAS) score, the Disability of Arm and Shoulder (Quick DASH) score, and the American Shoulder and Elbow Surgeons (ASES) score were assessed for each patient upon their initial admission and again at the first year postoperative follow-up.

Statistical analyses

Continuous variables are reported as mean± standard deviation, as well as median and interquartile range (IQR) values, while categorical variables are presented as frequencies and percentages. The Shapiro-Wilk test was used to assess the normal distribution of continuous variables. In group comparisons, variables with normal distribution were analyzed using the independent two-sample t-tests, while the variables without normal distribution were analyzed using the Mann-Whitney U test. The analysis of categorical variable ratios was conducted utilizing the chi-square test. Additionally, the Wilcoxon signed-rank test was used to scrutinize the differences between repeated measurements.

A p-value of <0.05 was considered statistically significant in all analyses. Statistical Package for the Social Sciences (IBM SPSS Statistics for Windows, IBM Corp., Version 21, Armonk, NY) and NCSS v.21.0.3 (NCSS, LLC, Kaysville, UT, USA) software were used in the analyses.

## Results

Group 1, comprising 40 patients without acromioplasty, had an average age of 59.45±10.43 years. Among them, 27 (67.5%) were female. The 12 patients had comorbidities. These diseases were as follows: diabetes mellitus (n: 4), hypertension (n: 3), hypothyroidism (n: 3), and coronary artery disease (n: 2). The mean symptom duration was 10.4±4.3 months, and the mean follow-up period was 16.2±1.9 months. Group 2, which underwent acromioplasty, included 45 patients with an average age of 56.49±8.97 years, with 30 (66.7%) of them being female. The 11 patients had comorbidities. These diseases were as follows: diabetes mellitus (n: 5), hypertension (n: 2), hypothyroidism (n: 2), and coronary artery disease (n: 2). The mean symptom duration was 9.5±3.6 months, and the mean follow-up time was 15.78±2.17 months in this group.

The mean preoperative abduction degrees were 79.25±13.08 and 84.89± 15.9 in Group 1 and Group 2, respectively. At the final follow-up, these angles were 114.75±16.01 and 115.78±13.56 for Group 1 and Group 2, respectively. While other ROM values improved significantly after surgery, there was no statistical difference between the two groups. At the first-year follow-up, both groups showed a significant decrease in mean VAS and Quick DASH scores, along with a significant increase in the mean ASES score. However, the differences in the mean clinical scores were not significant between the groups. In both groups, postoperative cSAD and sSAD values showed a statistically significant increase compared to the preoperative values. Notably, the improvement in cSAD and sSAD values in Group 2 showed a statistically significant difference compared to Group 1 (Tables 1, 2).

**Table 1:** Range of motion, clinical scores and radiological measurements of Groups 1 and 2 VAS: Visual analog scale, DASH: Disability of Arm and Shoulder, ASES: American Shoulder and Elbow Surgeons, SAD: Sub-acromial distance

Group 1					Group 2				
	Preoperative	Postoperative			Preoperative	Postoperative			
	Mean ± SD	Mean ± SD	Change (%)	p	Mean ± SD	Mean ± SD	Change (%)	p	
Flexion	144.75 ± 11.98	155.50 ± 9.594	7,427	<0.001	141.56 ± 10.435	153.78 ± 6.839	8,632	<0.001	
Internal Rotation	44.25 ± 11.068	58.75 ± 7.574	32,768	<0.001	45.33 ± 10.135	60 ± 6.030	32,363	<0.001	
External Rotation	54.5 ± 12.999	67.25 ± 12.401	23,395	<0.001	56.22 ± 12.301	68.67 ± 8.944	22,145	<0.001	
Abduction	79.25 ± 13.085	114.75 ± 16.011	44,795	<0.001	84.89 ± 15.901	115.78 ± 13.566	36,388	<0.001	
VAS	6.988 ± 0.537	1.15 ± 0.427	83.543	<0.001	6.899 ± 0.411	1.122 ± 0.372	83,737	<0.001	
Quick DASH	59.376 ± 5.571	21.463 ± 2.667	63,852	<0.001	57.554 ± 5.519	20.864 ± 2.639	63,749	<0.001	
ASES	21.395 ± 2.34	38.243 ± 2.939	78,747	<0.001	21.709 ± 1.944	38.589 ± 2.385	77,757	<0.001	
Coronal SAD	6.133 ± 0.784	6.488 ± 0.632	5.79	<0.001	6.198 ± 0.774	6.984 ± 0.564	12.69	<0.001	
Sagittal SAD	6.148 ± 0.774	6.415 ± 0.604	4.34	<0.001	6.216 ± 0.778	6.904 ± 0.570	11.07	<0.001	

**Table 2:** Range of motion, clinical scores and radiological measurements in preoperative and postoperative periods VAS: Visual analog scale, DASH: Disability of Arm and Shoulder, ASES: American Shoulder and Elbow Surgeons, SAD: Sub-acromial distance

	Preoperative			Postoperative		
	Group 1	Group 2		Group 1	Group 2	
	Mean ± SD	Mean ± SD	p	Mean ± SD	Mean ± SD	p
Flexion	144.75 ± 11.98	141.56 ± 10.435	0.240	155.50 ± 9.594	153.78 ± 6.839	0.336
Internal Rotation	44.25 ± 11.068	45.33 ± 10.135	0.459	58.75 ± 7.574	60 ± 6.030	0.367
External Rotation	54.5 ± 12.999	56.22 ± 12.301	0.326	67.25 ± 12.401	68.67 ± 8.944	0.357
Abduction	79.25 ± 13.085	84.89 ± 15.901	0.085	114.75 ± 16.011	115.78 ± 13.566	0.755
VAS	6.988 ± 0.537	6.899 ± 0.411	0.337	1.15 ± 0.427	1.122 ± 0.372	0.581
Quick DASH	59.376 ± 5.571	57.554 ± 5.519	0.022	21.463 ± 2.667	20.864 ± 2.639	0.295
ASES	21.395 ± 2.34	21.709 ± 1.944	0.351	38.243 ± 2.939	38.589 ± 2.385	0.551
Coronal SAD	6.133 ± 0.784	6.198 ± 0.774	0.548	6.488 ± 0.632	6.984 ± 0.564	0.337
Sagittal SAD	6.148 ± 0.774	6.216 ± 0.778	0.488	6.415 ± 0.604	6.904 ± 0.570	0.867

## Discussion

In the current study, we observed significant improvements in the clinical outcomes of patients undergoing arthroscopic RC repair, irrespective of whether acromioplasty was performed. While patients who received acromioplasty during RC repair demonstrated greater radiological improvement, there was no additional clinical benefit observed in this cohort.

RC syndrome is one of the common problems in orthopaedics and traumatology, with a growing global incidence [4,10]. Factors such as age, comorbid diseases, activity levels, and smoking are among the known risk factors for RC tears [4]. However, the relationship between acromion and glenoid morphology and RCTs, as well as their role in treatment, remains a topic of discussion in the literature [4,11-13]. Notably, Bigliani et al. reported a high incidence of RCTs in individuals with a hooked (type 3) acromion structure [14].

Subacromial pain syndrome and RC tears are thought to be associated with a reduction in the subacromial space [15,16]. Therefore, some have advocated for the routine inclusion of acromioplasty during RC repair procedures to increase this space [15]. Cheng et al. stated that the pain and ASES scores in patients who underwent acromioplasty were superior compared to those who underwent RC repair alone in a one-year follow-up study [16]. Similarly, another study indicated that individuals with type 3 acromion had poorer Constant, Simple Shoulder Test (SST), and VAS scores when compared to those with type 1 acromion [17]. Several studies have suggested that ROM may improve more significantly in patients who undergo acromioplasty for RCTs. Furthermore, there is an argument that performing acromioplasty may reduce the risk of RC re-injury [18]. In their study with an average follow-up of 11 years, Woodmass et al. reported higher reoperation rates among patients with types 2-3 acromion who had only undergone RC repair compared to those who had both RC repair and acromioplasty [19].

The necessity of acromioplasty has been a matter of debate in recent years. Singh et al. published a study in 2021 that found no significant difference between groups based on whether acromioplasty was performed or not [6]. Gartsman and O’Connor showed that acromioplasty did not provide any superiority in functional outcomes in individuals with full-thickness RC tears and type 2 acromions [20]. Similarly, Shin et al. demonstrated that acromioplasty had no effect on clinical outcomes [5]. In a study by Waterman et al. conducted after 7.5 years of follow-up, acromioplasty performed during RC repair showed no effect on clinical scores and revision rates [21]. Another study even indicated that concurrent acromioplasty with RC repair was associated with an increased rate of revision [22]. In Kolk et al.’s study, the researchers found no significant difference between the group that underwent only bursectomy and the group that underwent bursectomy and acromioplasty in patients operated on for subacromial pain syndrome, after an average of 12 years of follow-up [23].

SAD, also known as acromiohumeral distance, refers to the shortest distance between the undersurface cortex of the acromion and the top of the humeral head on a Grashey radiograph [24]. While this measurement was initially defined using radiography, it can now be obtained using ultrasound (USG) and MRI. The most critical difference between radiography and other measurement techniques lies in the patient’s position. Discussions regarding the impact of the dorsal decubitus position on MRI measurements and its potential alteration of results due to reduced gravitational effects continue in the literature [24]. In a study conducted by Mirzayan et al., it was stated that the SAD value was significantly smaller in MRI compared to AP radiographs in patients with RCT arthropathy [25]. Another study evaluating the relationship between RC injury and the elevation of the humeral head revealed that MRI evaluations remained unaffected by gravitational force, with the main factors being the size and shape of the tear as well as accompanying lesions [9].

Limitations of the study

This study had some limitations, most importantly its retrospective nature. The inclusion of only type 3 acromion patients restricted the sample size, and longer follow-up periods are necessary to assess reoperation rates for these individuals. On the other hand, the study's strength lies in its more objective evaluation, as it exclusively included patients with type 3 acromion and no additional pathologies, such as subscapularis tears. Unlike many similar studies employing different imaging modalities for measurements, this study exclusively utilized MRI.

## Conclusions

Our study exclusively included patients with type 3 acromion and evaluated the pre- and post-operative SAD values using MRI. While SAD increased in all patients undergoing RC repair, the acromioplasty group exhibited a greater increase. However, this did not translate into superior clinical outcomes. In conclusion, we observed significant improvements in the clinical outcomes of patients undergoing arthroscopic RC repair, irrespective of whether acromioplasty was performed. Although SAD increased in all RC repair patients, the acromioplasty group demonstrated a more substantial increase. While patients who received acromioplasty during RC repair demonstrated greater radiological improvement, concomitant acromioplasty had no significant effect on clinical outcomes.
